# Exploring the Evolutionary Relationship of Insulin Receptor Substrate Family Using Computational Biology

**DOI:** 10.1371/journal.pone.0016580

**Published:** 2011-02-25

**Authors:** Chiranjib Chakraborty, Govindasamy Agoramoorthy, Minna J. Hsu

**Affiliations:** 1 School of Bioscience and Technology, VIT University, Vellore, India; 2 College of Environmental and Health Sciences, Tajen University, Yanpu, Taiwan; 3 Department of Biological Sciences, National Sun Yat-Sen University, Kaohsiung, Taiwan; University of South Florida College of Medicine, United States of America

## Abstract

Insulin receptor substrate (IRS) harbors proteins such as IRS1, IRS2, IRS3, IRS4, IRS5 and IRS6. These key proteins act as vital downstream regulators in the insulin signaling pathway. However, little is known about the evolutionary relationship among the IRS family members. This study explores the potential to depict the evolutionary relationship among the IRS family using bioinformatics, algorithm analysis and mathematical models.

## Introduction

The discovery of insulin in 1922 symbolized a milestone in medicine and it has also contributed considerably to the progress in the field of molecular endocrinology. The significance of insulin used in the treatment of diabetes drew enormous interest in this hormone and scientists have been studying the mechanisms of insulin signaling proteins to understand how the cascading works at cellular level. In the insulin signaling process, insulin binds to the alpha subunit of the receptor that activates the tyrosine kinase in beta subunit [1], [2]. This process also starts autophosphorylation of several tyrosine residues present in the beta subunit [3]. They are recognized by phosphotyrosine-binding domains of adaptor proteins namely the insulin receptor substrate family (IRS) members [4]. The IRS protein cascades are the common elements in the peripheral response and signaling pathway since these protein cascades are recognized by others in the signaling pathway for further downstream action. It results ultimately in the uptake and storage of glucose as glycogen [5]. Therefore the insulin receptor substrate family serves as a key mediator not only in signaling but also in growth and function of pancreatic beta-cell [6], [7]. In case of a failure in the IRS cascade binding, it may cause hyperinsulinemia and peripheral insulin resistance [8].

The insulin receptor substrate 1 or IRS1 is known to be associated with the increase or decrease in blood glucose level. For example, liver-specific knockdown of IRS1 may leads to an up-regulation of gluconeogenic enzymes such as glucose 6 phosphatase (G6Pase) and phosphoenolpyruvate carboxy kinase (PEPCK). Reduction of IRS1 level in contrast may cause decline of glucokinase (GK) expression level, and may increase glucose levels in the blood [9], [10]. Reports indicate that the knockdown of IRS2 is responsible for the up-regulation of lipogenic transcription factor, and sterol regulatory element binding protein 1c (SREBP-1c). Such up-regulation plays a key role in the consequence of insulin including transcription of hepatic genes such as glucokinase and fatty acid genes [9], [10]. Research also shows that IRS3 and IRS4 can influence and change the actions of IRS1 and IRS2 [11]. Although these two protein cascades (IRS3 and IRS4) may not have the ability to activate MAPK and PI3K, they can antagonize the functions of IRS1 and IRS2 when expressed at high levels. Besides, scientists have demonstrated that IRS5 and IRS6 to have limited signaling function due to the expression of IRS5 mainly in kidney and liver, while IRS6 expressing more in skeletal muscles [12]. So far only six members have been isolated from the IRS-family; they are IRS1, IRS2, IRS3, IRS4, IRS5 and IRS6, respectively. However, studies are needed to understand their relationships [6], [12]. Some members such as IRS1 and IRS2 are widely distributed in the human body while others have restricted distribution (IRS3 in adipocytes and brain, IRS4 in embryonic tissues or cell lines, IRS5 in kidney and liver, and IRS6 in skeletal muscle) [6], [13].

Biological evolution involves genetic change in population and all organisms that exist now in our planet are based on the same fundamental genetic information encoded as nucleic acid transcribed into RNA, and then into proteins (polymers of amino acid) by highly conserved ribosome. Thus scientists can use amino acid sequences to predict the structural or functional regions of proteins by analyzing conservation patterns. In fact, these regions directly involve in biochemical functioning such as binding surfaces on the surface of proteins [14]. Scientists can also get additional information from protein glycosylation on protein folding, transport and function. Glycosylation plays a vital role in cell-cell interactions and antigenicity [15]. N-glycosylation and O-glycosylation are the two main types of glycosylation, and from them scientists can understand more on protein solubility, stability and structure. Such studies may yield new data on structural bioinformatics of protein [16], [17].

The evolutionarily conservation of a protein is positively correlated to the conservation positions of amino acid, which has structural and functional importance. Thus, conservation investigation of amino acid residue positions among members from the same family can reveal the importance of each position for the protein structure or function [18]. Therefore more scientific studies are needed to understand the conservation patterns of N-glycosylation sites and O-glycosylation sites of the IRS family members.

In this study, we have addressed this gap for the first time by performing a rapid structural bioinformatics analysis of the IRS family members. The comparative analysis was performed to obtain a better model of conservation patterns for the N-glycosylation and O-glycosylation sites. We have also illustrated a hypothetical structure of the IRS proteins with different protein binding domains and described the relationship among the IRS family members by using bioinformatics, algorithm analysis and mathematical models.

## Materials and Methods

### Data collection

We have collected data on genes related to proteins belong to the IRS family such as IRS1, IRS2, IRS4, IRS5 and IRS6 from the National Center for Biotechnology Information database (NCBI) [19]. The IRS3 gene was not available in the NCBI database, so we obtained it from the IRS3L pseudogene sequence for IRS3 gene. The functional protein sequences (in FASTA format) were gathered from the NCBI database and further analyzed.

### Multiple sequences alignment and generation of scores

The sequences were given to ClustalW for the multiple sequences alignment [20]. Based on the multiple sequence alignment techniques, we observed similarities in the sequences. We have used six sequences in our analysis and ClustalW (ver. 1.83) was used to elucidate respective alignment scores. IRS1, IRS2, IRS3L, IRS4, IRS5 and IRS6 sequences were represented as Seq1, Seq2, Seq3, Seq4, Seq5, and Seq6, respectively. We also used notation Seq (x:y) meaning alignment scores between sequence x, and sequence y, and the scores were applied further for analysis. Multiple sequence alignment (MSA) was finally merged into one by using profile to profile alignment MUSCLE [21]. All alignments used in this study have been provided separately.

### Phylogenetic tree construction

Based on the results of sequence alignments, we constructed the phylogenetic tree using a user-friendly computer software (Phylogeny.fr) and computational biology [22]. We have developed two types of phylogenetic tree namely phylogram and cladogram (excluding branch length), and the phylogram shows distances among protein sequences within the IRS family.

### Sequence logos of conserved domains

A sequence logo was formed using the WebLogo software to develop graphical representation of amino acid or nucleic acid and for displaying the patterns in a set of aligned sequences [23], [24]. We have used 53 amino acids from all sequences to visualize patterns of aligned sequences as well as bias amino acid sequences all within the IRS family.

### Conservation patterns and highly conserved amino acids

The conservation patterns of structures in IRS family members were formed using ConSurf server [18], [25]. The conservation scores at each amino acid position were calculated using the same server. We have calculated the evolutionary conservation of amino acid positions in proteins using an empirical Bayesian inference starting from protein structure and sequence. Highly conserved amino acids from proteins were used for further analysis.

### Glycosylation site prediction

Post translational modifications (PTMs) occur in vast majority of proteins and are essential for function [15], [26]. Prediction of the sequence location of PTMs enhances the functional characterization of proteins. Glycosylation is a type of PTM, which has been implicated in protein folding, transport and function. We have performed the prediction of N-glycosylation and O-glycosylation sites using the NetNglyc and NetOglyc glycosylation predictors [15], [26].

## Results and Discussion

**Figure 1:**
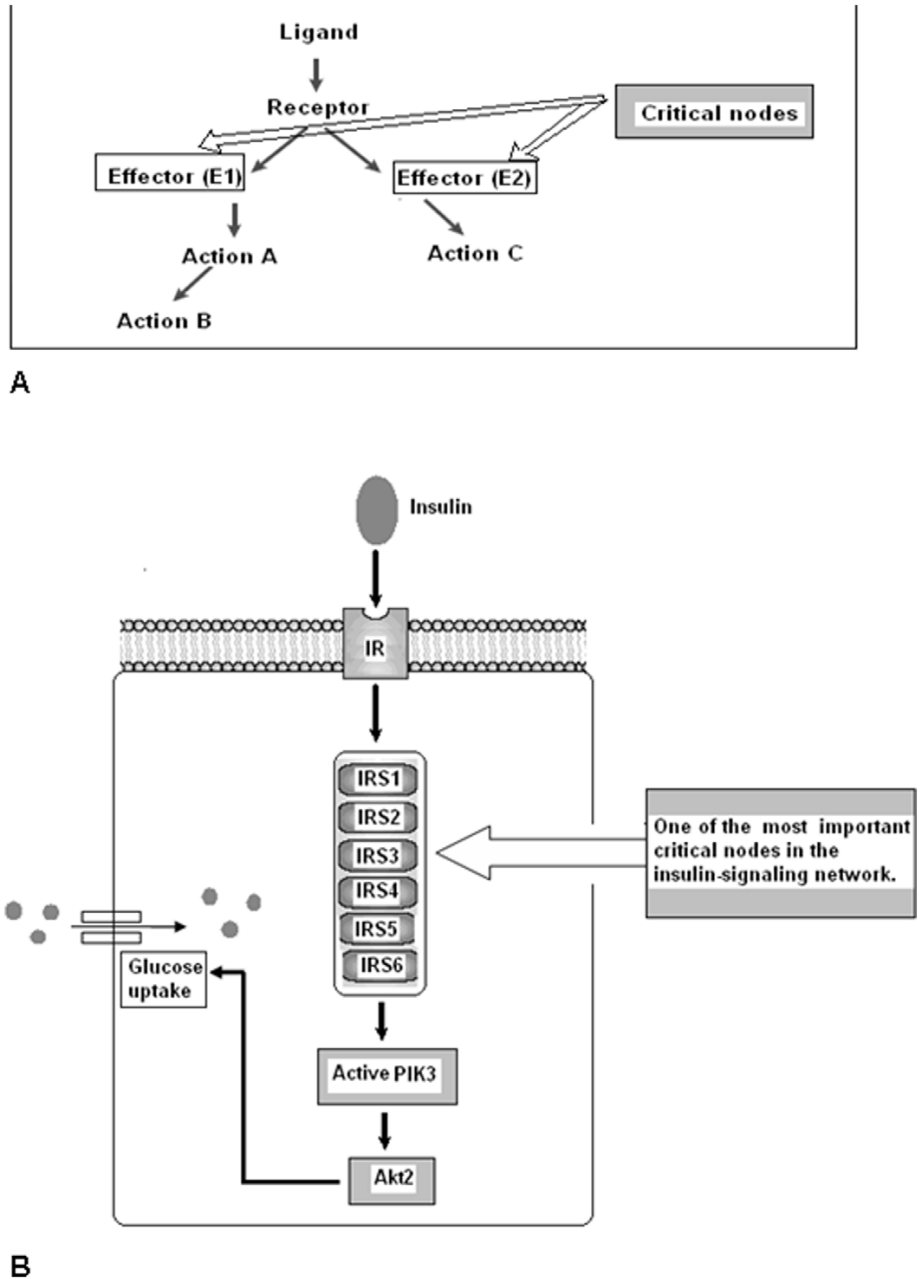
Critical node concept in the insulin signaling network. (A) Critical node is the nodal point which consists of the effect molecules for a further downstream action in a signaling pathway. (B) This pathway shows that IRS family members belong to a critical node. This node is one of the important node in a signaling path way.

The IRS family member proteins and their genes were recorded using original data derived from the NCBI data bank (Table S1). The human IRS family member proteins related to insulin signaling pathway (Figure 1) and their protein identification, accession number, GI and length of the protein were documented (Table S2). The result of the multiple sequence alignment (MSA) provided as Figure S1. Sequence alignment scores between the sequences were illustrated in Figure S2. Sequence alignment shows highest scores (56) between the sequences 5 (IRS5) and 6 (IRS6) thus matching the best. But, the lowest scores (04) were observed among the sequences 3 (IRS3) and 6 (IRS6).

**Figure 2:**
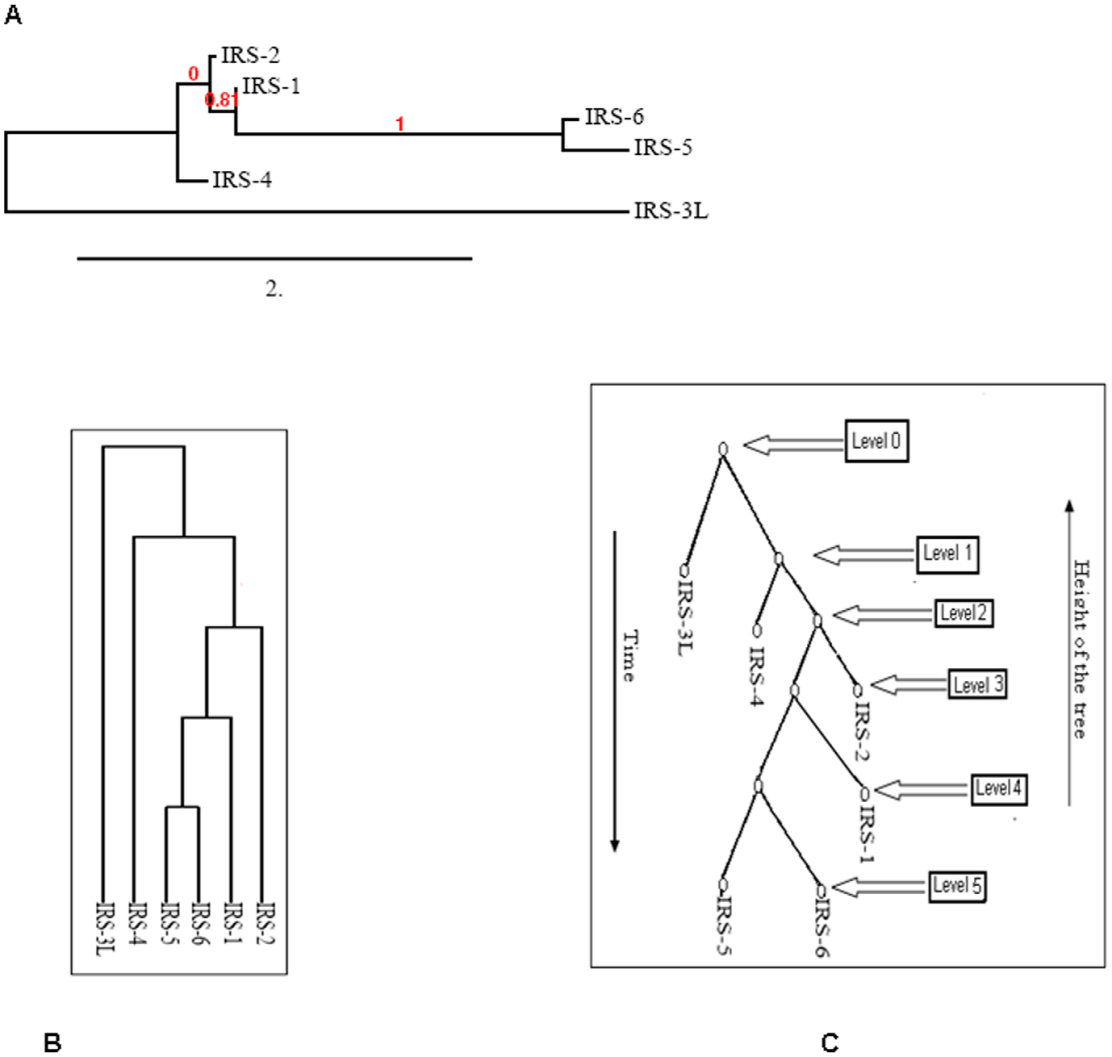
Phylogenetic tree construction. (A) Phylogram tree with the distances between the protein sequences of the IRS family members. Bootstrap support values are indicated at nodes. IRS protein family members names at the clade. (B) Cladogram of protein sequences of the IRS family members for tree algorithm analysis (C) Representation binary tree equivalent to Cladogram.

We have developed phylogram, cladogram and binary tree, which is equivalent to cladogram and our findings show significant relationships among the proteins in IRS family members (Figure 2A, 2B and 2C). The phylogenetic analysis of IRS family members was depicted using amino acid sequences of individual member proteins. In the phylogenetic tree, the distance of branches was developed from the likelihood ratio mapping the evolutionary relationships among distinct members of IRS family. While developing the tree algorithm, we have drawn another figure (Figure 2C) from the cladogram (Figure 2B) that shows clearly the phylogenetic tree rooted with ideal binary (Figure 2C). The rooted tree contains internal nodes and each internal node also contains two children nodes. The height of the binary tree level was 5.

**Figure 3:**
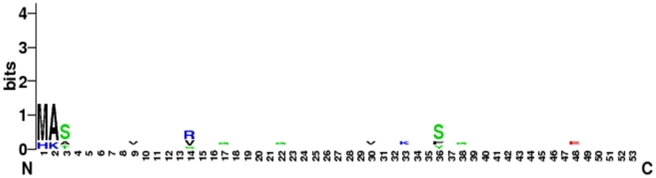
WebLogo for functional proteins associated with the IRS family members. Protein sequences using residues 1–53 fragment of the IRS at a time to generate the WebLogo.

We have also showed the graphical representation of amino acid for all functional proteins such as IRS1, IRS2, IRS3, IRS4, IRS5 and IRS6, respectively in Figure 3. Every logo consists of one letter or one stacks of letters for each position in the sequence. The height of each stack shows the sequence conservation at that position measured in bits. The height of symbols within the stack reveals the relative frequency of that subsequent amino acid at that particular position (positions like 1,2,3,9,14,17,22,30,33,36,48, and 1–3 and 36 contain more stack of amino acid with a maximum stack height of 1.4 bits, minimum height of 0.2 bits; Figure 3).

**Figure 4:**
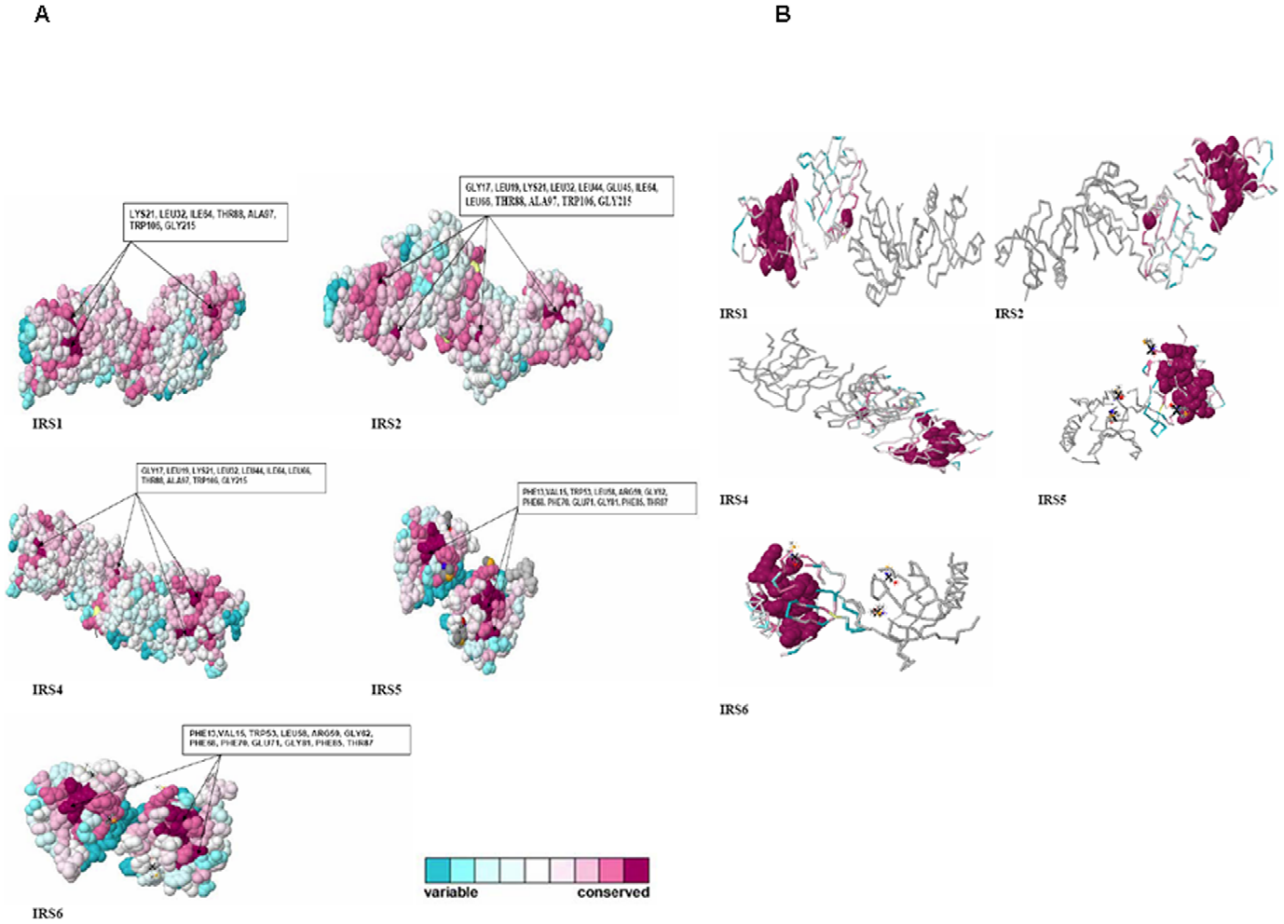
Conservation patterns and backbone structures of the proteins. (A) Shows the general conservation patterns with highly conserved amino acids in 3D structure of the IRS family members. Amino acid conservation scores were classified into 9 levels. The color scale for residue conservation is indicated in the figure. (B) Backbone structures with highly conserved amino acids of IRS family members proteins.

The conservation patterns of proteins in the IRS family and their backbone structures have been shown in Figure 4. However, IRS3L was excluded from the analysis since the software was not able to predict the conservation pattern accurately. Nonetheless, we successfully documented highly conserved amino acids of each protein (Figure 4). The highly conserved amino acids residues for IRS1 are LYS21, LEU32, ILE64, THR88, ALA97, TRP106, GLY215; for IRS2 are GLY17, LEU19, LYS21, LEU32, LEU44, GLU45, ILE64, LEU66, THR88, ALA97, TRP106, and GLY215. IRS4 showed conserved surface formed by residues GLY17, LEU19, LYS21, LEU32, LEU44, ILE64, LEU66, THR88, ALA97, TRP106, and GLY215. Similarly, IRS5 and IRS6 showed similar conserved residues such as PHE13, VAL15, TRP53, LEU58, ARG59, GLY62, PHE68, PHE70, GLU71, GLY81, PHE85, and THR87.

By reviewing the N-glycosylation sites (Figure S3), we were able to notice the following: IRS1 with 7 N-glycosylation sites (at the residue position of 275, 352, 370, 734, 742, 1076, 1082), IRS2 with 4 N-glycosylation sites (at the residue position of 28, 212, 768, 1179), IRS3L with 4 N-glycosylation sites (at the residue position of 320, 595, 847, 874), IRS4 with 4 N-glycosylation sites (at the residue position of 183, 724, 773, 1191), and IRS5 with 1 N-glycosylation site (at the residue position of 4), respectively. The N-glycosylation sites were absent in IRS6. While reviewing the O-glycosylation sites (Figure S4), we found the following: IRS1 with 242 O-glycosylation sites, IRS2 with 230 sites, IRS3 with 240 O-glycosylation sites, and IRS4 with 171 O -glycosylation sites, respectively. On the other hand, IRS5 was found with 48 while IRS6 had 41 O-glycosylation sites.

With more understanding of the proteome, we are in the process of knowing the complexities involved in cell-signaling networks, especially the critical nodes, which form an important part of the signaling network that functions downstream of the insulin receptor and growth factor. The concept of critical nodes or key signaling nodes in a signaling pathway is the budding perception [13], [27], [28]. We have adopted the critical node concept and analyzed the members' critical node in a signaling pathway (Figure 1). In the complex insulin signaling pathway, IRS node is crucial where the IRS family members incorporate and function downstream with the insulin receptor. However, studies have shown the importance of the critical node concept in the insulin-signaling network and IRS family members indeed belong to critical node, and thus it complements our computational analysis [13]. The IRS proteins are cytoplasmic proteins and they function as essential cascades for downstream signaling. They have highest level of homology in their N-termini. These proteins share two extremely conserved domains, which are pleckstrin homology (PH) domain and phosphotyrosine binding (PTB) domain. The former is responsible for protein-protein interactions plus protein-phospholipid interactions while the latter is accountable for the interactions with NPXY motifs in activated receptors [29], [30]. The IRS-proteins have multiple tyrosine phosphorylation motifs in the COOH-terminal portion while IRS-1 and IRS-2 show about 35% similarity in this region. However, this similarity is restricted to potential tyrosine phosphorylation sites [31]. At least eight tyrosines on IRS-1 undergo phosphorylation by the activated insulin receptor that includes residues 608, 628, 939 and 987. They occur in YMXM motifs [29]. In IRS2, the kinase regulatory loop binding domain has been identified while IRS-4 has PTB and PH domains, which binds to INSR. But they lack tyrosine phosphorylation and XYPPX motifs like other IRS [10], [30], [32]. Some common motifs have been found in IRS5 and IRS6 that include PTB, PH, cAMP phosphorylation, CK2phosphorylation, PKC phosp- horylation, myristoylation, and andtyrosine phosphorylation and these are similar to other IRS [9], [33], [34].

Using the multiple sequences alignment, we have generated scores on amino-acid matches and mismatches. The server used the substitution matrix to describe the rate at which how one character in a sequence changes to other character over time [35]. We have noted two scores greater than 30 (56 and 39). The alignment score 56 was generated between sequences 5 (IRS5) and 6 (IRS6) while score 39 was generated between sequences 1 (IRS1) and 2 (IRS2). We also found similarities among IRS5 and IRS6, and IRS1 and IRS2, respectively. However, lowest score (4) was observed between sequences 3 (IRS3) and 6 (IRS6) and it also showed dissimilarity.

We have constructed a phylogenetic tree to study the relationships between distinct members within the IRS family and noticed significant evolutionary relationship among its members. Our result shows that IRS5 and IRS6 have a common origin in evolutionary history. On the other hand, IRS1 and IRS2 seem to have lesser distance of branching length from the node. In general, the cladogram on n species (IRS family members) has 2n-1 edges and number of search Q(n) for any proteins in a cladogram tree in the range of log n≤Q(n)≤n ( where n = number of nodes in a binary tree) [36], [37]. From the computational complexity point of view, at level 0, one node is possible, and at level 1, mostly two nodes are possible, and so on. Hence the maximum number of nodes for binary tree at p level should be 2^0^+2^1^+2^2^+………+2^p^≥n. In a binary tree, length of the path between two leaf nodes determines the relationship. In the case of IRS5 and IRS6, the path lengths are closely related.

In recent years, a number of amino acid sequences are available in databases for free access. In addition, free availability of software further promotes the potential to assess conservation patterns of protein structure using computational biology. As a result, it is possible to study the evolution and divergence of paralogous and orthologous proteins. In this paper, we have showed the conserved amino acids in 3D structure proteins of IRS. Conservation pattern of insulin receptor family was also determined [38]. The conservation scores showed the evolutionary rate of a particular site of a protein, and some parts of the proteins evolve rapidly. They are commonly called as ‘variable’ and the positions in which they evolve slowly are called ‘conserved’. For example, the IRS5 and IRS6 have more conserved residues than other proteins, while the conserved positions remain similar.

Most proteins undergo some form of post-translational modification (PTM), which is important for functionality [39]. Glycosylation is a well-known PTM, which plays a crucial role in protein folding and interactions with other molecules. Glycosylation, specially N-glycosylation and O-glycosylation provide structural and functional information about the proteins. Our result shows that IRS family members have highly glycosylated protein comprising both N- and O-linked glycosylation sites. The members in fact have more O-glycosylation regions than N-glycosylation. During correct folding of any substrate proteins, the O-glycosylation process influence different parameters of substrate protein folding [40]. Studies have shown that increase in O-glycosylation sites of the IRS1 and IRS2 as well as some other insulin signaling proteins HBP activation condition [41].

This study has demonstrated a rapid comparative and structural bioinformatics analysis of insulin receptor substrate family members. We have obtained a precise model of molecular phylogenetics, and conservation patterns of proteins with their N-glycosylation and O-glycosylation sites. Although some data are available for the insulin receptor substrate proteins [10], [30], this study presents new evidence on the evolutionary relationship among the insulin receptor substrate proteins. Using the latest bioinformatic tools supported by algorithm analysis and mathematical models, we have demonstrated that IRS5 and IRS6 are more closely related proteins than previously thought.

### Conclusions

In this work, we have applied an innovative and rapid approach to study the structural, functional and phylogenetic relationship among the insulin receptor substrate proteins. Our study shows a rapid way to calculate amino acid sequences in terms of evolutionary conservation rates and provides vital information about regions of structural and functional importance. The study demonstrates evolutionary conserved domains of IRS members with a strong selective process amongst the IRS members, which suggests that the conserved domains may have unknown significant physiological role in the insulin signaling pathway conserved from IRS1 to IRS6.

## Supporting Information

Figure S1 **Multiple Sequence Alignments (MSA) of proteins in IRS family members.**
(DOC)

Figure S2 **Alignment scores of protein sequences related to IRS isoforms.** (**A**) **A**lignment score between sequences (notation Seq (x:y) meaning alignment score between sequence x, and sequence y); (B) Scatter distribution of scores; (C) scores connected by smoothed line without marker.
(DOC)

Figure S3 **N-glycosylation of proteins of IRS family members.** (A)IRS1, (B)IRS2, (C)IRS3, (D)IRS4, (E)IRS5, and (F)IRS6.
(DOC)

Figure S4 **O-glycosylation sites of proteins of IRS family members.** (A)IRS1, (B)IRS2, (C)IRS3, (D)IRS4, (E)IRS5, and (F)IRS6.
(DOC)

Table S1 **Insulin receptor substrate proteins and their genes.**
(DOC)

Table S2 **Functional proteins associated with insulin resistance (** ***Homo sapiens*** **) and their protein IDs analyzed in this study.**
(DOC)
