# Supplementary material for: Exploring the Evolutionary Relationship of Insulin Receptor Substrate Family Using Computational Biology
Source: PLoS One. 2011 Feb 25;6(2):e16580. doi: 10.1371/journal.pone.0016580 (PMC3045367; doi:10.1371/journal.pone.0016580)
Supplement: Figure S1 — Multiple Sequence Alignments (MSA) of proteins in IRS family members. (DOC) [file pone.0016580.s001.doc]

IRS-1 ------------------------------------------------------------

IRS-2 ------------------------------------------------------------

IRS-5 ------------------------------------------------------------

IRS-6 ------------------------------------------------------------

IRS-4 ---------------------MASCSFTRDQATRRLRGAAAAAAAALAAVVTTPLLSSGT 39

IRS-3L HKASLPSLVPPPPRQEAQEECGQGLTWRSDGGGGIFFKSVPCGTALPWALASAVLLMDGS 60

IRS-1 -------MASPPESD--------------------GFSDVRKVGY--------------- 18

IRS-2 -------MASPPRHGPPGPASGDGPNLNNNNNN--NNHSVRKCGY--------------- 36

IRS-5 -------MAT-------------------------NFSDIVKQGY--------------- 13

IRS-6 -------MAS-------------------------NFNDIVKQGY--------------- 13

IRS-4 PTALIGTGSSCPGAMWLSTATGSRSDSESEEEDLPVGEEVCKRGY--------------- 84

IRS-3L AVGKVGERGTVLMGQPDSPETQKAQVAQVLLVAQLGQHTLSSCSTPCRGPPLFCFPSAQD 120

.: : . .

IRS-1 ------LRKPKSM---HKRF------------FVLRA----------------------- 34

IRS-2 ------LRKQKHG---HKRF------------FVLRG----------------------- 52

IRS-5 ----VKMKSRKLG--IYRRCW-----------LVFRK----------------------- 33

IRS-6 ----VRIRSRRLG--IYQRCW-----------LVFKK----------------------- 33

IRS-4 ------LRKQKHG---HRRY------------FVLKL----------------------- 100

IRS-3L ERFTIELTRARLLSSTLVSCWCHVWIP---VFYIYRGFSNIAYYIIDLSICTYIHIYTHP 177

: : : :

IRS-1 ------------------------ASEAGG----PARLEYYENEKKWRHKSSA------- 59

IRS-2 -----------------PGAGGDEATAGGGSAPQPPRLEYYESEKKWRSKAGA------- 88

IRS-5 ------------------------SSSKG-----PQRLEKYPDEKSVCLRGCP------- 57

IRS-6 ------------------------ASSKG-----PKRLEKFSDERAAYFRCYH------- 57

IRS-4 ------------------------ETADA-----PARLEYYENARKFRHSVRAAAAAAAA 131

IRS-3L ADLPLPDCSRMWGMGIPICASTIKAGAPGLEGGQPCVLTP-TIPRAWVFQGKALCNCLVF 236

. * * :

IRS-1 ------------PKRSIPLESCFNINKRADSKNKH-----------------LVALYTRD 90

IRS-2 ------------PKRVIALDCCLNINKRADAKHKY-----------------LIALYTKD 119

IRS-5 --------------KVTEISNVKCVTRLPKETKRQ----------------AVAIIFTDD 87

IRS-6 --------------KVTELNNVKNVARLPKSTKKH----------------AIGIYFNDD 87

IRS-4 AASGAAIPPLIPPRRVITLYQCFSVSQRADARYRH-----------------LIALFTQD 174

IRS-3L FFFFFSPCASLSPNLCTKAHCRQCWKRTAFERKRKRRLWFCFRPLGVAGQSTLILDLALL 296

: . : :

IRS-1 EHFAIAADSEA--EQDSWYQALLQLHNRAKGHHDG-AAALGAGGGGGSCSGS------SG 141

IRS-2 EYFAVAAENEQ--EQEGWYRALTDLV--SEGRAAA-GDAPPAAAPAASCSASLPGALGGS 174

IRS-5 SARTFTCDSEL--EAEEWYKTLSVECLGSRLNDIS-LGEPDLLAPGVQCEQT-------- 136

IRS-6 TSKTFACESDL--EADEWCKVLQMECVGTRINDIS-LGEPDLLATGVEREQS-------- 136

IRS-4 EYFAMVAENES--EQESWYLLLSRLILESKRRRCGTLGAQPDGEPAALAAAA-------- 224

IRS-3L GWTAWASDNAAIGQRQGRARPCLNKSPALAMRRSV-AGEEPPLPGTGVCWMGECPLGPGW 355

: ..:. : : .

IRS-1 LGEAGEDLSYGDVPPG-PAFKEVWQVI---LKPKGLGQTKNLIGIYRLCLTSKTIS---- 193

IRS-2 AGAAGAEDSYGLVAPATAAYREVWQVN---LKPKGLGQSKNLTGVYRLCLSARTIG---- 227

IRS-5 ---------------------DRFNVF---LLP---CPNLDVYGECKLQITHENIY---- 165

IRS-6 ---------------------ERFNVY---LMP---SPNLDVHGECALQITYEYIC---- 165

IRS-4 ----------AAEPPF---YKDVWQVI---VKPRGLGHRKELSGVFRLCLTDEEVV---- 264

IRS-3L AKEVGANVVLEVDPSW------VWNILCPRLFPPGDVVTTSSCGVWAICVVTRQTWSGGG 409

::: : * . * : : .

IRS-1 -------------------------------------------------FVKL-N----- 198

IRS-2 -------------------------------------------------FVKL-N----- 232

IRS-5 -------------------------------------------------LWDIHN----- 171

IRS-6 -------------------------------------------------LWDVQN----- 171

IRS-4 -------------------------------------------------FVRL-N----- 269

IRS-3L WQTPHLAGQRPPCCLAQGQVRPLPFHHCPGPVAIHSWGLSESSTESSKEIWELGDQCIYF 469

: : :

IRS-1 ------------SEAAAVVLQLMNIRRCGHSENFFFIEVGRSAVTG-----------PGE 235

IRS-2 ------------CEQPSVTLQLMNIRRCGHSDSFFFIEVGRSAVTG-----------PGE 269

IRS-5 ------------PRVKLVSWPLCSLRRYGRDATRFTFEAGRMCDAG-----------EGL 208

IRS-6 ------------PRVKLISWPLSALRRYGRDTTWFTFEAGRMCETG-----------EGL 208

IRS-4 ------------TEVASVVVQLLSIRRCGHSEQYFFLEVGRSTVIG-----------PGE 306

IRS-3L ADFSGNNLLLLFARLGLASLSFPTIRTPSCLFLFFSQPLPSFCLLGGLPLRSWPLHVPSL 529

. : :* . * * .

IRS-1 FWMQVDDSVVAQNMHETILEAMRAMSD--EFRPRSKSQSS-SNCSNPISVP--LRRHHLN 290

IRS-2 LWMQADDSVVAQNIHETILEAMKALKELFEFRPRSKSQSSGSSATHPISVPGARRHHHLV 329

IRS-5 YTFQTQE---GEQIYQRVHSATLAIAE--------------------------------- 232

IRS-6 FIFQTRD---GEAIYQKVHSAALAIAE--------------------------------- 232

IRS-4 LWMQVDDCVVAQNMHELFLEKMRALCAD-EYRARCRSYSI-SIGAHLLTLLSARRHLGLV 364

IRS-3L LFLPSSPPIISVSLYLILLPVLSFLLR--------------AGGREAGICYGVLGGGAFL 575

: . :: . :

IRS-1 NPPPSQVGLTRRSRTESITATSPASMVGGKPGSFRVRASSDGEGTM----------SRPA 340

IRS-2 NLPPSQTGLVRRSRTDSLAATPPAA----KCSSCRVRTASEGDGGAAAGAAA--AGARPV 383

IRS-5 ----QHKRVLLEMEK--------------------------------------------- 243

IRS-6 ----QHERLLQSVK---------------------------------------------- 242

IRS-4 PLEPG--GWLRRSRFEQF---------------CHLRAIGDGEDEMLF--------TRRF 399

IRS-3L LFPPSFHHPSLNARSAEV-WNGTEGLSPASVTLCCLPASPHYLGP---------GPGPPC 625

.

IRS-1 SVDGSPVSPSTN----RTHAHRH-------------------------------RGSARL 365

IRS-2 SVAGSPLSPGPV----RAPLSRSHTLSGG----------------------CGGRGSKVA 417

IRS-5 ----------------NVRLL--------------------------------------- 248

IRS-6 ----------------NSMLQ--------------------------------------- 247

IRS-4 VTPSEPVAHSRRG---RLHLPRG-------------------------------RRSRRA 425

IRS-3L RLPPLQPTRTPACWLVRAGAVRSRQ---------------------------VSLGPQGS 658

.

IRS-1 HPP----LNHSRSIPMPASRCSPSATSPVSLSSS-STSGHGSTSDCLFPRRSS------- 413

IRS-2 LLPAGGALQHSRSMSMPVAHSPPAATSPGSLSSS-SGHGSGSYPPPPGPHPPLPHPLHHG 476

IRS-5 ---------------NKGTEHYSYPCTPTTM----------------LPRS--------- 268

IRS-6 ---------------MKMSER---AASLSTMVP--------------LPRS--------- 266

IRS-4 VSVPASFFRRLAPSPARPRHPAEAPNNGARLSSEVSGSGSGNFGEEGNPQGKEDQEGSGG 485

IRS-3L LGTGHELLSLAGDSSSSSGSSPPVSRLSWRVAKNGERPGLRGNGENIAWLQIQTHHTHHG 718

. :

IRS-1 -------------------ASVSGSPSDGGFISSDEYGSSPCDFR--SSFRSVTPDSLGH 452

IRS-2 P-----GQR-----PSSGSASASGSPSDPGFMSLDEYGSSPGDLRAFCSHRSNTPESIAE 526

IRS-5 ------------------------------------------------------------

IRS-6 ------------------------------------------------------------

IRS-4 D-----YMPMNNWGSGNGRGSGGGQGSNGQGSSSHSSGGNQCSGEGQGSRGGQGSNGQGS 540

IRS-3L ARVTHLGSHQLLANAGTMLCRQQSSSMAPAFSQSVTCGPSPCVRKQESATKCLHIGACGS 778

IRS-1 TPPARGEE---ELSNYICMGGKGPSTLTAPNGHYILSRG-GNGHRCTPGTGLGTSPALAG 508

IRS-2 TPPARDGGGGGEFYGYMTMDRP-------------LSHC-GRSYRRVSGD---------- 562

IRS-5 -----------AYWHHIT------------------------------------------ 275

IRS-6 -----------AYWQHITRQHS-------------------------------------- 277

IRS-4 GGNQCSRDGQGTAGGHGSGGGQRPGGGHGSGGGQGPGDGHGSGGGKNSGGGKGSG---SG 597

IRS-3L DLWARGWEQGGGLNVWLC------------------------------------------ 796

IRS-1 DEAASAADLDNRFRKRTHSAGTSPTITHQKTPSQSSVASIEEYTEMMPAYPPGGGSGGRL 568

IRS-2 ----AAQDLDRGLRKRTYSLTTP---ARQRPVPQPSSASLDEYTLMRATFS---GSAGRL 612

IRS-5 ------------------------------------------------------------

IRS-6 ------------------------------------------------------------

IRS-4 KGSDGDGERGKSLKKRSYFGKLTQSKQQQMPPPPPPP----------PPPPPAGGTGGKG 647

IRS-3L ----PCVAFHRGARPQAEEGGARWNSLVSSPWIPPNPHSSIGAENAVPRPQGKVNPSGQE 852

IRS-1 PG--HRHSAFVPTR------------------------SYPEEGLEMHPLERRG------ 596

IRS-2 CPSCPASSPKVAYH------------------------PYPEDYGDIEI----------- 637

IRS-5 ------------------------------------------------------------

IRS-6 ------------------------------------------------------------

IRS-4 KSGGRFRLYFCVDRGATKE------------------CKEAKEVKDAEIPEGAAR----- 684

IRS-3L RQSGSATSCPW------------------------------------------------- 863

IRS-1 ------------------------------------------------------------

IRS-2 ------------------------------------------------------------

IRS-5 ------------------------------------------------------------

IRS-6 ------------------------------------------------------------

IRS-4 ------------------------------------------------------------

IRS-3L ------------------------------------------------------------

IRS-1 ------------------------------------------------------------

IRS-2 ------------------------------------------------------------

IRS-5 ------------------------------------------------------------

IRS-6 ------------------------------------------------------------

IRS-4 ------------------------------------------------------------

IRS-3L ------------------------------------------------------------

IRS-1 ------------------------------------------------------------

IRS-2 ------------------------------------------------------------

IRS-5 ------------------------------------------------------------

IRS-6 ------------------------------------------------------------

IRS-4 ------------------------------------------------------------

IRS-3L ------------------------------------------------------------

IRS-1 ----------------------------GHHRPDSSTLHTDDGYMPMSPGVAPVPSGRKG 628

IRS-2 ----------------------------GSHRSSSSNLGADDGYMPMTPGAALAGSGSGS 669

IRS-5 ----------------------------GSQNIAEASSYAGEGY-----GAAQASS---- 298

IRS-6 ---------------------------------------TGQLY-----RLQDVSSPL-- 291

IRS-4 ----------------------------GPHRARAFDEDEDDPYVPMRPGVATPLVS--- 713

IRS-3L ----------------------------GATQVTRAT--GNKSFPGRVKGSVLPRQLMAS 893

.. :

IRS-1 --SGDYMPMSPKSVSAPQQIINPIRRHP-----QRVDPNGYMMMSPSGGCSPDIGGGPSS 681

IRS-2 CRSDDYMPMSPASVSAPKQILQPRAAAAAAAAVPSAGPAGPAPTSAAGRTFPASGGG-YK 728

IRS-5 ------------------------------------------------------------

IRS-6 ------------------------------------------------------------

IRS-4 --SSDYMPMAPQNVSASKK----RHSRS-----PFEDSRGYMMMFPRVSPPPAPSPPKAP 762

IRS-3L CVTPGLSWYSVGPITWHSPGIDQHPPHPGLPSPQALRSLLFLPFLPMCHPAPKVQHRVVW 953

IRS-1 SSSSSNAVPSGTSYGKLWTNGVGGHHSHVLPHPKPPVESSGGKLLPCTGDYMN------- 734

IRS-2 ASSPAESSPEDSGYMRMWCG------------SKLSMEHADGKLLP-NGDYLN------- 768

IRS-5 ------------------------------------------------------------

IRS-6 ------------------------------------------------------------

IRS-4 DTNKEDDSKDNDS----------------------------------ESDYMF------- 781

IRS-3L SWSLSQLFHKEGCCRGTWLMGSG---------------KGKERDLPQVSSSLR------- 991

IRS-1 -----MSPVGDSNTSSPSDCYYGPEDPQ-------HKPVLSYYSLPRSFKHTQRPGEPEE 782

IRS-2 -----VSPSDAVTTGTPPDFFSAALHPG--GEPLRGVPGCCYSSLPRSYKAPYTCG---- 817

IRS-5 ------------ETDLLNRFILLKPKPS-------------------------------- 314

IRS-6 -----------------------KLH---------------------------------- 294

IRS-4 -----MAPGAGAIPKNPRNPQGGSSSKS--W--------SSYFSLPNPFRSSPLGQ---- 822

IRS-3L -----EKRLLGPECSRDRGPLVASLRQQ--QDWEGEGCAHVLCVPAPLHMVPVSRPLATW 1044

IRS-1 GARHQHLRL--STSSGRLLYAATAD----DSSSSTSSDSLGGGYCGARLEPSLPHPHHQV 836

IRS-2 GDSDQYVLM--SSPVGRILEEERL-----EPQATPGPSQAASAF---GAGPTQP-PHPVV 866

IRS-5 ------------------------------------------------------------

IRS-6 ------------------------------------------------------------

IRS-4 NDNSEYVPMLPGKFLGRGLDKEVSYN--WDPKDAASKPSGEGSFSKPGDG-----GSPSK 875

IRS-3L GYGLEHSPVLLATYGAIASVANTQHWAVAEPRPTAPPPHTTPPVLRASPAPQWGFGLPEV 1104

IRS-1 --LQPHLPR--KVDTAAQTNSRLARPTRLSLGDPKASTLPRAREQQQQQQPLLHPPEPKS 892

IRS-2 --PSPVRPSGGRPEGFLGQRGRAVRPTRLSLEG--LPSLPSMHE-----YPL--PPEPKS 915

IRS-5 ------------------------------------------------------------

IRS-6 ------------------------------------------------------------

IRS-4 --PSDHEPP----------KNKAKRPNRLSFITKGYKIKPKPQK------PTHEQREADS 917

IRS-3L TWSRPWVPP----ACWLGAGWFPASPSQCDL---GIGVGPQLHA-HSTCLPYSHSRGAPS 1156

IRS-1 PGEYVNIEFGSDQSGYLSGPVA------------------------FHSSPSVRCPSQLQ 928

IRS-2 PGEYINIDFG-EPGARLSPPAP-------------------PLLASAASSSSLLSASSPA 955

IRS-5 ------------------------------------------------------------

IRS-6 ------------------------------------------------------------

IRS-4 SSDYVNMDFTKRES---NTPAP--------------------------STQGLPDSWGII 948

IRS-3L PGSRKCGGKPCPPCSGCPGPWARFG-----SLHLFSCSVFVISCSFPEGAPPMPSLWGLH 1211

IRS-1 PAPREEETGT---------------EEYMKMDLG----PGRRAAWQEST----GVEMGRL 965

IRS-2 SSLGSGTPGTSSDSRQRSP-----LSDYMNLDFSSPKSPKPGAPSGHPV----GSLDGLL 1006

IRS-5 ------------------------------------------------------------

IRS-6 ------------------------------------------------------------

IRS-4 AEPRQSAF-----------------SNYVNVEFGVP-FPNPANDLSDLL--------RAI 982

IRS-3L VGLSSLTLGFPLAPYLPWVISQGPLRSHQVSPAGLSGAPSPPSPWRGSVVLGSELSAKMS 1271

IRS-1 G-----------------PAPPGAASICRPTRAVPSSRGDYMTMQMSCPRQSYVDTSPAA 1008

IRS-2 SPEASSPYPPLP------PRPSASPSSSLQPPPPPPAPGELYRLPPASAVATAQGPGAAS 1060

IRS-5 ------------------------------------------------------------

IRS-6 ------------------------------------------------------------

IRS-4 P-----------------RANPLSLDSARWPLPP---------LPLSATGSNAIE----- 1011

IRS-3L SPQPNRNFFKKQ------RVPPGSPHVHGQSLQVVPGYFWAVVARRGPEQWGSPKGSSEM 1325

IRS-1 PVS--------YADMRTGIAAEEVSLPRATMAAASSSSAASASPTGPQGAAELAAHSSLL 1060

IRS-2 SLSSDTGDNGDYTEMAFGVAA---TPPQPIAAPPKPEAARVASPTSGVKRLSLMEQVS-- 1115

IRS-5 ------------------------------------------------------------

IRS-6 ------------------------------------------------------------

IRS-4 -------EEGDYIEVIFNSAM---TPAMALA----DSAIRYDAETGRIYVVDPFSECC-- 1055

IRS-3L ASSSEVSFSGHLTGFQRVWSVHRDYPLSLLSPSHLLSPQTFKKLNLQLRNNPAQCSVAVA 1385

IRS-1 GGPQGPGGMSAFTRVNLSPNRNQSAKVIRADPQGCRRRHS-SETFS------------ST 1107

IRS-2 -------GVEAFLQASQPPDPHRGAKVIRADPQGGRRRHS-SETF--------SSTTTVT 1159

IRS-5 ----------------------------QGDSSEAKTPSQ-------------------- 326

IRS-6 ----------------------------RTETFPAYRSEH-------------------- 306

IRS-4 --------MDISLSPSRCSEPPPVARLLQEEEQERRRPQSRSQSFFAAARAAVSAFPTDS 1107

IRS-3L PLVTWKGTKPLLDGKGVHTGRTPTGRAGRGRSQQGAWRPR-SKALSPAPPPLPQVRSLAS 1444

:

IRS-1 P---------------------------SATRVGNTVPFGAGAAVGGGGGS----SSSSE 1136

IRS-2 PVSPSFAHNPKRHN------------SASVENVSLRKSSEGGVGVGPGGGD----EPPTS 1203

IRS-5 ------------------------------------------------------------

IRS-6 ------------------------------------------------------------

IRS-4 LERDLSPSSAPAVAS------------AAEPTLALSQVVAAASALAAAPGI----GAAA- 1150

IRS-3L HRGFLGCPDDVSGPQ------------SADRRRPHALCPYKGSVLGSRPPLLDFPTQTPV 1492

IRS-1 DVKRHSSASF---ENVWL--RPGELGGAPKEPAKLCG--AAGGLENGLNYIDLDLVKDFK 1189

IRS-2 PRQLQPAPPLAPQGRPWTPGQPGGLVGCPGSGGSPMRRETSAGFQNGLNYIAIDVREE-- 1261

IRS-5 ------------------------------------------------------------

IRS-6 ------------------------------------------------------------

IRS-4 -----AAAGFDSASARWF--QP--VANAADAEAVRGAQDVAGGSNPGAHNPSANLARGDN 1201

IRS-3L PGRREDSRGVGVPERSAEGGRWGAQARGNSHRDTRCAQSPCPKLPVGSPGETLGIKTGGS 1552

IRS-1 QCPQECTPEPQPPPPP------PPHQPLGSGESSSTR----------------------- 1220

IRS-2 ---PGLPPQPQPPPPP----LPQPGDKSSWGRTRSLGGLISAVGVG----------STGG 1304

IRS-5 ------------------------------------------------------------

IRS-6 ------------------------------------------------------------

IRS-4 QAGGAAAAAAAPEPPPRSRRVPRP------------------------------------ 1225

IRS-3L LPGRCSRPAEFATSPPPLLASPRAQDTRPSRLCLFCSHFIAFAPESPQAAAPG---RAGN 1609

IRS-1 ----RSSEDLS----AYASISFQKQPEDRQ------------ 1242

IRS-2 GCGGPGPGALP-PANTYASIDFLSHHLKEATIVKE------- 1338

IRS-5 ------------------------------------------

IRS-6 ------------------------------------------

IRS-4 ----PEREDSDNDDDTHVRMDFARRDNQFDSPKRGR------ 1257

IRS-3L GRRAPQPAAALGLPCRRAVLRPPARRPAASRVFRX------- 1644

**Figure S1. Multiple Sequence Alignments (MSA) of proteins in IRS family members.**
