# Supplementary material for: Exploring the Evolutionary Relationship of Insulin Receptor Substrate Family Using Computational Biology
Source: PLoS One. 2011 Feb 25;6(2):e16580. doi: 10.1371/journal.pone.0016580 (PMC3045367; doi:10.1371/journal.pone.0016580)
Supplement: Figure S2 — Alignment scores of protein sequences related to IRS isoforms. (A) Alignment score between sequences (notation Seq (x:y) meaning alignment score between sequence x, and sequence y); (B) Scatter distribution of scores; (C) scores connected by smoothed line without marker. (DOC) [file pone.0016580.s002.doc]

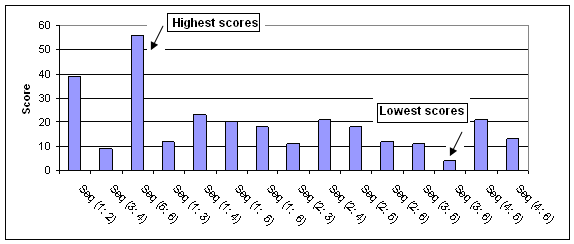


**A**


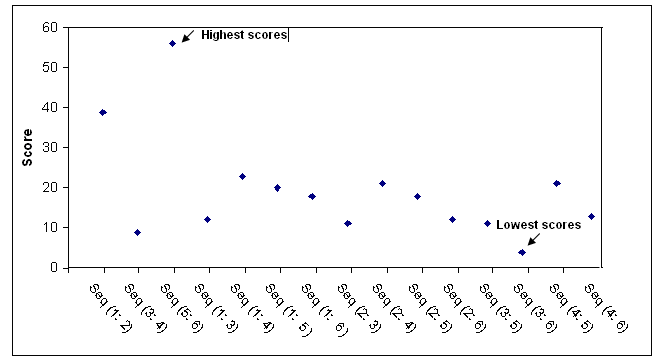


**B**

**C**

**Figure S2. Alignment scores of protein sequences related to IRS isoforms.**

1. alignment score between sequences (notation Seq (x:y) meaning alignment score between sequence x, and sequence y); (B) Scatter distribution of scores; (C) scores connected by smoothed line without marker.
