# Supplementary material for: Exploring the Evolutionary Relationship of Insulin Receptor Substrate Family Using Computational Biology
Source: PLoS One. 2011 Feb 25;6(2):e16580. doi: 10.1371/journal.pone.0016580 (PMC3045367; doi:10.1371/journal.pone.0016580)
Supplement: Figure S3 — N-glycosylation of proteins of IRS family members. (A)IRS1, (B)IRS2, (C)IRS3, (D)IRS4, (E)IRS5, and (F)IRS6. (DOC) [file pone.0016580.s003.doc]

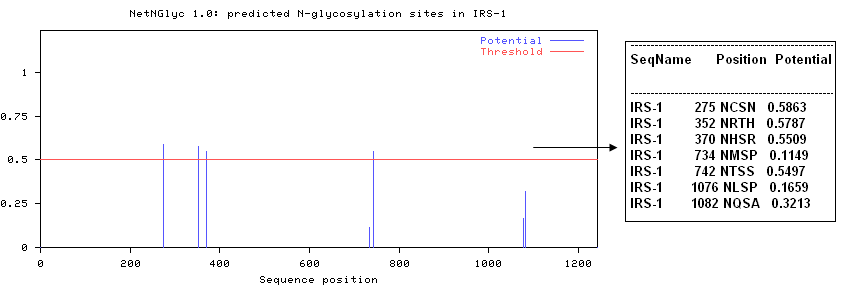


**(A)IRS1**

**
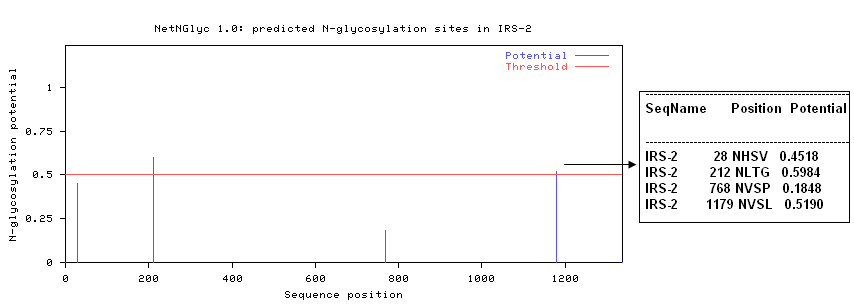
**

**(B)IRS2**

**
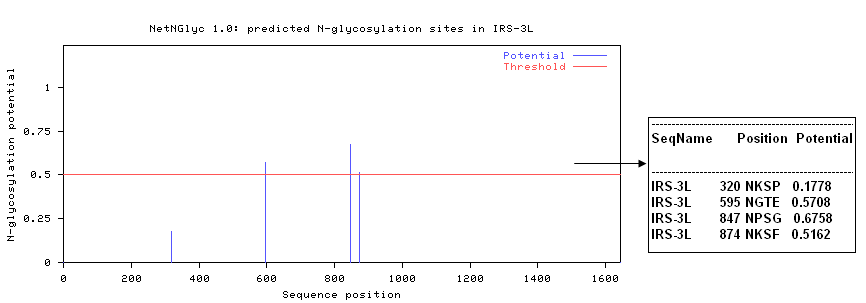
**

**(C)IRS3L**

**
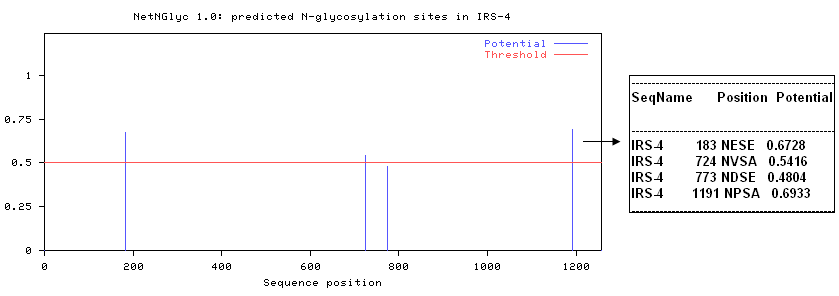
**

**(D)IRS4**

**
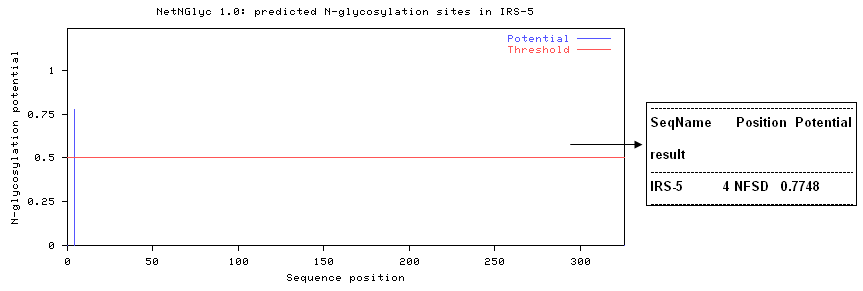
**

**(E)IRS5**

**
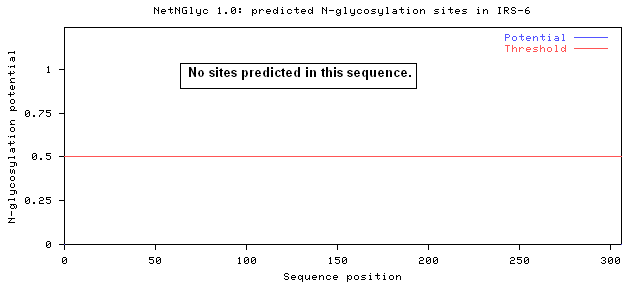
**

**(F)IRS6**

**Figure S3. N-glycosylation of proteins of IRS family members.** (A)IRS1, (B)IRS2, (C)IRS3, (D)IRS4, (E)IRS5, and (F)IRS6.
