# Supplementary material for: Exploring the Evolutionary Relationship of Insulin Receptor Substrate Family Using Computational Biology
Source: PLoS One. 2011 Feb 25;6(2):e16580. doi: 10.1371/journal.pone.0016580 (PMC3045367; doi:10.1371/journal.pone.0016580)
Supplement: Figure S4 — O-glycosylation sites of proteins of IRS family members. (A)IRS1, (B)IRS2, (C)IRS3, (D)IRS4, (E)IRS5, and (F)IRS6. (DOC) [file pone.0016580.s004.doc]

**
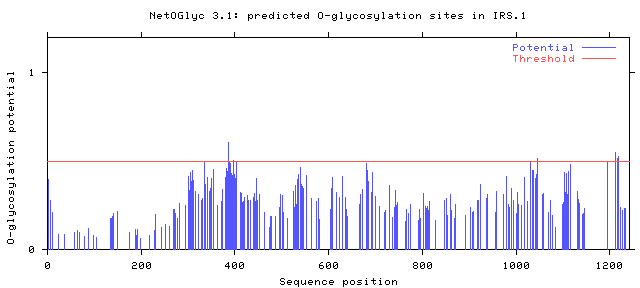
**

(A)IRS1


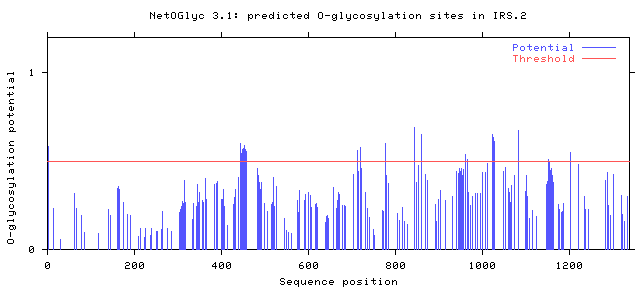


(B)IRS2


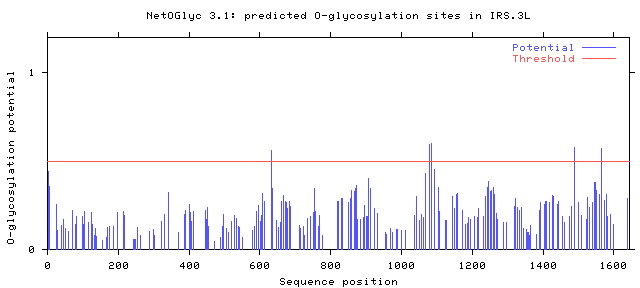


(C)IRS3


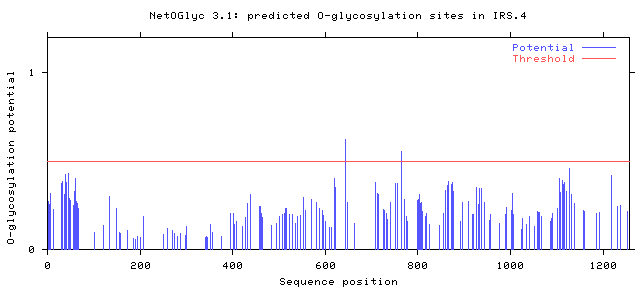


(D)IRS4


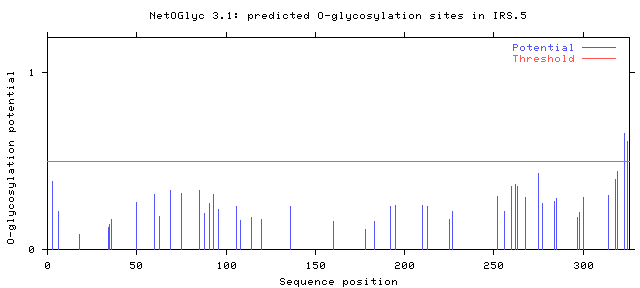


(E)IRS5


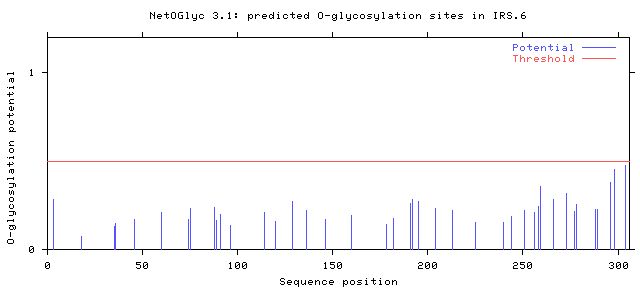


(F)IRS6

**Figure S4. O-glycosylation sites of proteins of IRS family members.** (A)IRS1, (B)IRS2, (C)IRS3, (D)IRS4, (E)IRS5, and (F)IRS6.
