# Supplementary material for: Exploring the Evolutionary Relationship of Insulin Receptor Substrate Family Using Computational Biology
Source: PLoS One. 2011 Feb 25;6(2):e16580. doi: 10.1371/journal.pone.0016580 (PMC3045367; doi:10.1371/journal.pone.0016580)
Supplement: Table S1 — Insulin receptor substrate proteins and their genes. (DOC) [file pone.0016580.s005.doc]

**Table S1.** **Insulin receptor substrate proteins and their genes.**

| **No** | **Gene/Locus** | **Gene Location** | **Protein(Gene product)** | **Function** | **Reference** |
| --- | --- | --- | --- | --- | --- |
| **1** | *IRS1* | ****Chromosome:****2; ****Location:**** 2q36 | Insulin receptor substrate 1 | Insulin receptor substrate 1 plays a key role in transmitting signals from the insulin and insulin receptors to intracellular pathways PI3K kinase / Akt and Erk MAP kinase pathways. | Bommer  et al.^42^ |
| **2** | *IRS2* | ****Chromosome:****13; ****Location:**** 13q34 | Insulin receptor substrate 2 | This gene encodes the insulin receptor substrate 2, a cytoplasmic signaling molecule that mediates effects of insulin | Verdier et al.^43^ |
| **3** | *IRS3L* | ****Chromosome:**** 7; ****Location:**** 7q22.1 | Insulin receptor substrate 3 | IRS-3 has been identified only in rodents*.* Therefore, *IRS3L* has been chosen which can encode the insulin receptor substrate 3 like protein^46^. However, another study ^44^ showed that RS-3 is localized to both cytosol and nucleus, and possesses transcriptional activity and also identified Bcl-3 as a novel binding protein to IRS-3. IRS-3 enhanced NF-kappaB-dependent anti-apoptotic gene induction and consequently inhibited TNF-alpha-induced cell death. It has been proposed that a novel function for IRS-3 as a transcriptional regulator in TNF-alpha signaling, distinct from its function as a substrate of insulin/IGF receptor kinases. | Kabuta et al.^44^ |
| **4** | *IRS4* | ****Chromosome:**** X; ****Location:**** Xq22.3 | Insulin receptor substrate 4 | IRS-4 Mediates Protein Kinase B Signaling during Insulin Stimulation. IRS-4 also promoted the activation of PKB/Akt and BAD phosphorylation during insulin stimulation | Uchida et al.^45^ |
| **5** | *IRS-5/*  *DOK4* | ****Chromosome:**** 16; ****Location:**** 16q21 | Insulin receptor substrate 5 | Insulin receptor-phosphorylated IRS5/DOK4 associates with RasGAP, Crk, Src, and Fyn, but not phosphatidylinositol 3-kinase p85, Grb2, SHP-2, Nck, or phospholipase Cgamma Src homology 2 domains, and activates MAPK in cells. IRS5/DOK4 has a potential roles in insulin and IGF-1 action. | Cai et al.^12^ |
| **6** | *IRS-6/*  *DOK5* | ****Chromosome:**** 20; ****Location:**** 20q13.2 | Insulin receptor substrate 6 | IRS6/DOK5 is a signaling protein and having potential roles in insulin and IGF-1 action. | Cai et al.^12^ |
