# Supplementary material for: Exploring the Evolutionary Relationship of Insulin Receptor Substrate Family Using Computational Biology
Source: PLoS One. 2011 Feb 25;6(2):e16580. doi: 10.1371/journal.pone.0016580 (PMC3045367; doi:10.1371/journal.pone.0016580)
Supplement: Table S2 — Functional proteins associated with insulin resistance ( Homo sapiens ) and their protein IDs analyzed in this study. (DOC) [file pone.0016580.s006.doc]

**Table S2.** **Functional proteins associated with insulin resistance (*Homo Sapiens)* and their protein IDs analyzed in this study**.

| Seq No | Protein | Protein/ gene ID | Other Information | Length |
| --- | --- | --- | --- | --- |
| 1 | Insulin receptor substrate 1 | NP_005535.1 | LOCUS NP_005535  DEFINITION insulin receptor substrate 1 [Homo sapiens].  ACCESSION NP_005535  VERSION NP_005535.1 GI:5031805 | 1242 aa |
| 2 | Insulin receptor substrate 2 | NP_003740.2 | LOCUS NP_003740  DEFINITION insulin receptor substrate 2 [Homo sapiens].  ACCESSION NP_003740  VERSION NP_003740.2 GI:38683860 | 1338 aa |
| 3 | Insulin receptor substrate 3-like | (NG_005538.4)  HGNC:6127 | LOCUS NG_005538  ( pseudogene) DEFINITION Homo sapiens insulin receptor substrate 3-like (IRS3L) | 1644 aa |
| 4 | Insulin receptor substrate 4 | GenBank: EAX02682.1 | LOCUS EAX02682  DEFINITION insulin receptor substrate 4 [Homo sapiens].  ACCESSION EAX02682  VERSION EAX02682.1 GI:119623087 | 1257 aa |
| 5 | Insulin receptor substrate 5 (or Downstream of kinase 4/docking protein 4) | Swiss-Prot: Q8TEW6.2 | LOCUS DOK4_HUMAN  DEFINITION RecName: Full=Docking protein 4; AltName: Full=Downstream of tyrosine kinase 4; AltName: Full=Insulin receptor substrate 5; Short=IRS-5; Short=IRS5.  ACCESSION Q8TEW6  VERSION Q8TEW6.2 GI:84029600 | 326 aa |
| 6 | Insulin receptor substrate 6 (or Downstream of kinase 5/docking protein 5) | Swiss-Prot: Q9P104.2 | LOCUS DOK5_HUMAN  DEFINITION RecName: Full=Docking protein 5; AltName: Full=Downstream of tyrosine kinase 5; AltName: Full=Insulin receptor substrate 6;  Short=IRS-6; Short=IRS6.  ACCESSION Q9P104  VERSION Q9P104.2 GI:26393190 | 306 aa |
